# WSX-1 Signalling Inhibits CD4^+^ T Cell Migration to the Liver during Malaria Infection by Repressing Chemokine-Independent Pathways

**DOI:** 10.1371/journal.pone.0078486

**Published:** 2013-11-07

**Authors:** Ana Villegas-Mendez, Emily Gwyer Findlay, J. Brian de Souza, Lisa-Marie Grady, Christiaan J. Saris, Thomas E. Lane, Eleanor M. Riley, Kevin N. Couper

**Affiliations:** 1 Department of Immunology and Infection, London School of Hygiene and Tropical Medicine, London, United Kingdom; 2 Faculty of Life Sciences, University of Manchester, Manchester, United Kingdom; 3 Division of Infection and Immunity, University College London, London, United Kingdom; 4 Department of Inflammation Research, Amgen, Inc., Thousand Oaks, California, United States of America; 5 School of Biological Sciences, University of California Irvine, Irvine, California, United States of America; University of California, Riverside, United States of America

## Abstract

IL-27 is an important and non-redundant regulator of effector T cell accumulation in non-lymphoid tissues during infection. Using malaria as a model systemic pro-inflammatory infection, we demonstrate that the aberrant accumulation of CD4^+^ T cells in the liver of infected IL27R^−/−^ (WSX-1^−/−^) mice is a result of differences in cellular recruitment, rather than changes in T cell proliferation or cell death. We show that IL-27 both inhibits the migratory capacity of infection-derived CD4^+^ T cells towards infection-derived liver cells, but also suppresses the production of soluble liver-derived mediator(s) that direct CD4^+^ T cell movement towards the inflamed tissue. Although CCL4 and CCL5 expression was higher in livers of infected WSX-1^−/−^ mice than infected WT mice, and hepatic CD4^+^ T cells from WSX-1^−/−^ mice expressed higher levels of CCR5 than cells from WT mice, migration of CD4^+^ T cells to the liver of WSX-1^−/−^ mice during infection was not controlled by chemokine (R) signalling. However, anti-IL-12p40 treatment reduced migration of CD4^+^ T cells towards infection-derived liver cells, primarily by abrogating the hepatotropic migratory capacity of T cells, rather than diminishing soluble tissue-derived migratory signals. These results indicate that IL-27R signalling restricts CD4^+^ T cell accumulation within the liver during infection primarily by suppressing T cell chemotaxis, which may be linked to its capacity to repress Th1 differentiation, as well as by inhibiting the production of soluble, tissue-derived chemotaxins.

## Introduction

The IL-12 cytokine superfamily member IL-27 is an important regulator of pro-inflammatory immune responses [1], [2]. Increased numbers of effector CD4^+^ T cells are found in the livers of IL-27R (TCCR/WSX-1) deficient mice during *Plasmodium berghei* NK65, *Toxoplasma gondii*, *Leishmania donovani* and *Trypanasoma cruzi* infections [3]–[6], in the lungs of WSX-1^−/−^ mice during *Mycobacterium tuberculosis* infection and in the intestine of WSX-1^−/−^ mice during *Trichuris muris* infection [7]–[9]. The mechanisms through which IL-27 limits Th1, Th2 and Th17 responses, enhances CD4^+^ T cell IL-10 production and regulates the polarisation of Foxp3^+^ regulatory T cells, have been widely studied [1], [2]. By contrast, the pathways by which IL-27 inhibits effector T cell accumulation in non-lymphoid tissues during infection are poorly understood, but may include limiting CD4^+^ T cell proliferation or enhancing cellular apoptosis *in situ*.

In support of these theories, CD4^+^ T cells from WSX-1^−/−^ mice are hyper proliferative following *in vitro* stimulation with anti-CD3/CD28 [6], [10] and proliferate more extensively in the lungs of WSX-1^−/−^ mice than WT mice during *Mycobacterium tuberculosis* infection [9]. Whilst the role of IL-27 in controlling T cell apoptosis has not been directly examined, IL-6 and IL-12 are both known to exert anti-apoptotic effects on CD4^+^ T cells [11], [12] and concentrations of both these cytokines are significantly increased in WSX-1^−/−^ mice during infection [9], [10], [13], [14]. Alternatively, IL-27 might limit the autonomous chemotactic activity of CD4^+^ T cells, and/or affect the expression of liver derived chemotactic/migratory factors. In support of this latter hypothesis, we have shown that splenic CD4^+^ T cells from malaria-infected WSX-1^−/−^ mice express higher levels of CCR5 than cells from WT mice and are consequently hyper responsive to CCR1 and CCR5 ligands [15].

In this study we have used *Plasmodium berghei* NK65 as a model systemic infection, to investigate the pathways by which IL-27 restricts effector CD4^+^ T cell accumulation in the liver during inflammation. We demonstrate that intrahepatic CD4^+^ T cell proliferation and apoptosis are unaffected by the absence of WSX-1 signalling. Instead our results show that IL-27 attenuates CD4^+^ T cell accumulation in the liver by inhibiting T cell migratory pathways. Surprisingly, we find that CD4^+^ T cell accumulation in the livers of infected WSX-1^−/−^ mice is not orchestrated by non-classical chemokine pathways. Rather, increased CD4^+^ T cell migration in infected WSX-1^−/−^ mice seems to result from the loss of IL-27-mediated suppression of Th1 differentiation and chemotaxis. We conclude that IL-27 restricts the accumulation of pathogenic T cells in the liver during infection by co-ordinately suppressing soluble, non-chemokine, chemotactic signals and by repressing the development of highly migratory Th1 cells. These results expand our understanding of how IL-27 signalling regulates tissue inflammation and opens up new avenues of research into how T cells enter inflamed tissues.

## Materials and Methods

### 1. Ethics Statement

All animal work was approved following local ethical review by LSHTM and University of Manchester Animal Procedures and Ethics Committees and was performed in strict accordance with the U. K Home Office Animals (Scientific Procedures) Act 1986 (approved H.O Project Licenses 70/6995 and 70/7293).

### 2. Mice and Parasites

C57BL/6 mice were purchased from Harlan, UK. Breeding pairs of C57BL/6 IL-27R deficient (WSX-1^−/−^) mice [10] were provided by Amgen Inc (Thousand Oaks, USA). Animals were maintained under barrier conditions in individually ventilated cages. Cryopreserved *P. berghei* NK65 parasites were passaged once through C57BL/6 mice before being used to infect experimental animals.

6–10 week old mice were infected by intravenous injection of 10^4^ parasitized red blood cells (pRBC). The course of infection was followed by monitoring weight loss and peripheral parasitaemia every 2^nd^ day. Parasitaemia was assessed by examination of Giemsa-stained thin-blood smears.

In some experiments, 250 µg anti-IL-12p40 (C17.8, BioXCell Inc, West Lebanon, USA) was injected i.p. every other day or 250 µg anti-CCL5 (R6G9, provided by Professor T Lane, University of California) or 250 µg TAK779 (obtained through the NIH AIDS Research and Reference Reagent Program, Division of AIDS, NIAID, NIH) were injected i.p. daily starting on day 7 post-infection.

### 3. Flow Cytometry

Single cell suspensions of spleen and livers were prepared by homogenising through a 70 µm cell strainer (BD Biosciences). Red blood cells (RBCs) were lysed using RBC lysis buffer (BD Biosciences). Leukocytes were enriched from liver homogenates by resuspending cell pellets in a 32% Percoll 3% 10× PBS 65% HBSS solution and centrifuging at 930 g for 10 minutes. Floating hepatocytes were removed and the cell pellet was collected. Absolute live cell numbers were calculated by microscopy and trypan blue exclusion.

Phenotypic characterisation of cell populations was performed according to previously published protocols [14], [15]. Cells were surface stained with: anti-mouse CD4 (clone GK1.5), CD44 (IM7), CD62L (MEL-14), CCR1 (PA1-41062), CCR4 (2G12), CCR5 (7A4), CCR7 (4B12), CXCR3 (CXCR3-173), CXCR4 (2B11), CXCR6 (221002), and KLRG1 (2F1) followed by intracellular staining of T-bet (4B10), Gata3 (TWAJ), foxp3 (FJK-16s), Ki67 (SolA15) and chemokine receptors. All antibodies were purchased from eBioscience or BD Biosciences.

For the analysis of cell proliferation *in vivo*, 1.25 mg sterile BrdU (5-bromodeoxyuridine) was injected ip. one hour before sacrifice. Intracellular BrdU incorporation was measured using an anti-BrdU antibody (clone PRB-1, eBioscience). T cell apoptosis was assessed using Annexin V (BD biosciences).

All flow cytometry acquisition was performed using an LSR II (BD Systems, UK). All analysis was performed using Flowjo Software (Treestar Inc, OR, USA). Fluorescence minus one controls were used to validate flow cytometric data.

### 4. Real Time PCR

RNA was extracted from livers (RNeasy, Qiagen) and DNAse I treated (Ambion, Texas, USA) before cDNA synthesis. mRNA levels of chemokine genes were quantified in whole liver tissue by real time PCR (Taqman) using validated gene expression assays from ABI Biosystems (Warrington, UK). cDNA expression for each sample was standardised to the housekeeping gene beta-actin. Data are presented as fold change (log_10_) in gene expression in infected tissues relative to uninfected tissues. Cycling conditions were: initialisation 2 min at 50°C and 10 min at 95°C followed by 40 cycles of 15 sec at 95°C and 1 min at 60°C.

### 5. Transwell Migration Assays

Migration of purified CD4^+^ T cells was assessed by their movement through a 5 µm pore transwell plate (Costar). 5×10^6^ cells from whole liver single cell suspensions were plated in the outer well of the transwell, and incubated at 37°C and 5% CO_2_ for one hour. Splenic CD4^+^ T cells were purified by magnetic bead separation (Miltenyi Biotec), labelled with 5 µM CFSE for 3 minutes (Molecular Probes, Invitrogen), washed twice with HBSS and then 1×10^6^ CD4^+^ T cells were plated in each inside well; cells were then incubated at 37°C and 5% CO_2_ for a further three hours. For analysis of cell migration towards the liver cells, cells in the outer well were collected and run on the flow cytometer to enumerate CFSE^+^ cells. For analysis of CD4^+^ T cell migration towards recombinant CCL4, CCL5 or CXCL12 (R&D Systems, Abingdon, UK or Peprotech, London, UK) all at 100 ng/ml), the number of cells that had migrated into the outer well was counted by light microscopy. The optimal concentrations of chemokines were determined in dose-response assays (results not shown).

In some migration assays chemokine inhibitors were used. In these experiments, purified CD4^+^ T cells were isolated and incubated with 100 ng/ml pertussis toxin (Sigma), 10 µg/ml anti-CCL5 or 1 µg/ml TAK779 or AMD3100 for two hours. Cells were then washed and treated as above. In cases where anti-CCL5 was used, liver cell suspensions were also incubated with 10 µg/ml anti-CCL5 during the one hour pre-assay incubation.

### 6. Adoptive Transfer Experiments

On day 10 post-infection mice were sacrificed and splenic CD4^+^ T cells isolated as described above. Cells were labelled with CFSE and incubated with either 100 ng/ml pertussis toxin or PBS for two hours at 37°C. Cells were then counted and 7×10^6^ of either the untreated or treated cells were injected i–v into recipient, infected (day 10 PI) mice. Mice were sacrificed on day 13 PI and CFSE^+^ cells in the liver were enumerated by flow cytometry.

### 7. Histology and Immunofluorescence

Livers were removed from uninfected and malaria-infected (D14 PI) mice and fixed in 10% formalin saline. Fixed tissues were paraffin embedded and sectioned before being stained with H&E. For immunofluoresence, sections were deparaffinization and dehydrated prior to antigen retrieval. Samples were incubated overnight with anti-CD45 (IBL-3/16) and anti-CD3 (145-2C11) primary antibodies (BD and AbD Serotec), washed, and incubated for an hour with Alexa flour 488 donkey anti rat and Alexa flour 488 goat anti hamster secondary antibodies (Life Technologies). Slides were examined under ×20 magnification using a Leica DMLB microsystem with CoolSnap camera and software.

### 8. Statistics

Statistical significance was determined using a Student’s T-test or one-way ANOVA with Tukey’s post hoc analysis. Results were classified as significantly different when P<0.05. All data are representative of 3 independent experiments.

## Results

### 1. Enhanced Accumulation of CD4^+^ T Cells in the Liver of Malaria-infected WSX-1^−/−^ Mice is not a Result of Altered *in situ* Proliferation or Apoptosis

**Figure 1:**
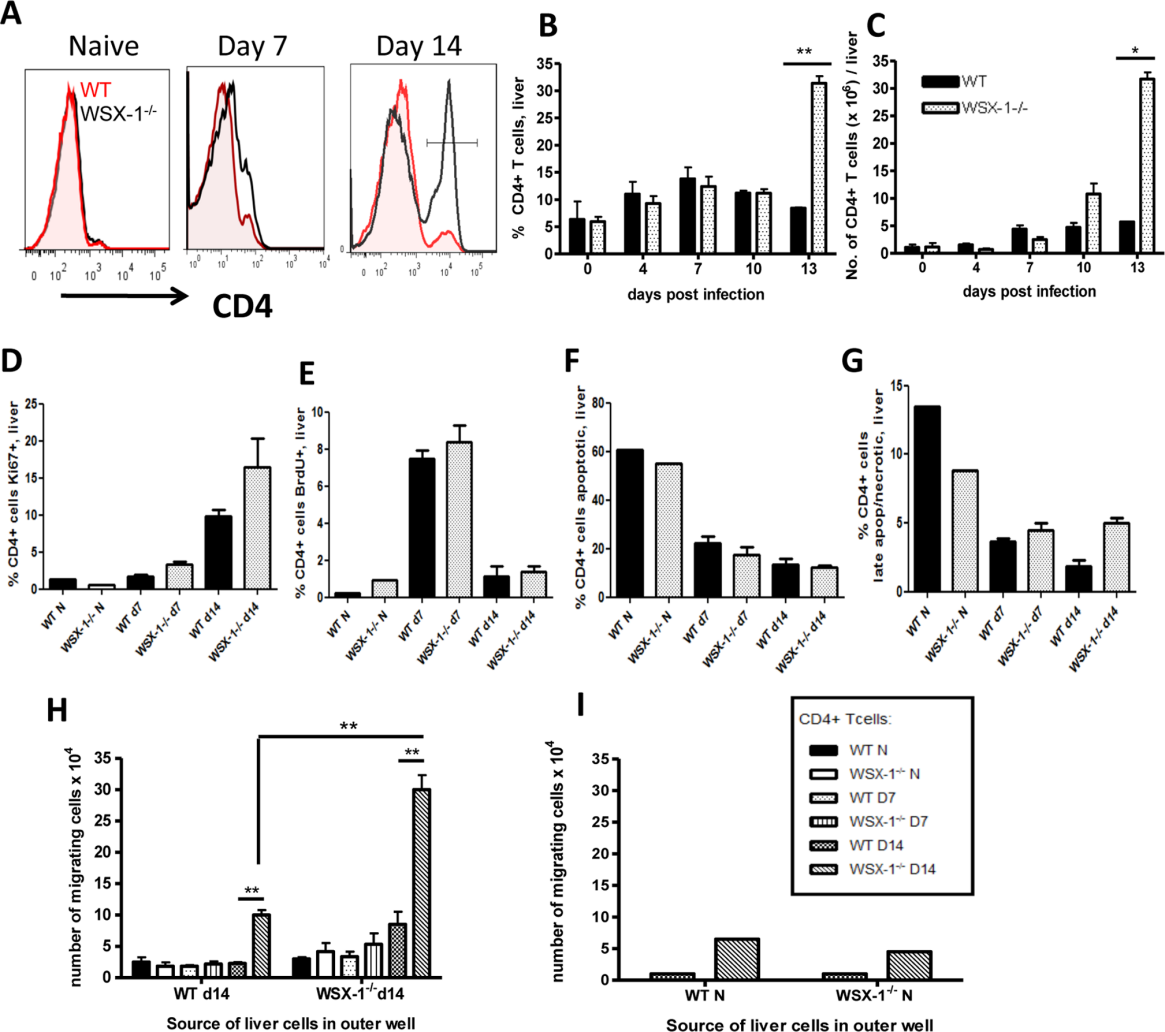
Accumulation of CD4^+^ T cells in the liver of WSX-1^−/−^ mice during malaria infection is caused by increased migration. (A) Representative Histograms, (B) frequencies and (C) numbers of intrahepatic CD4^+^ T cells in the liver of WT and WSX-1^−/−^ mice during infection. (D–G) The mean frequencies of intrahepatic CD4^+^ T cells expressing (D) BrDU, (E) Ki67 (F) annexin V^+^/7-AAD^−^ and (G) annexin V^+^/7-AAD^+^. (H–I) The migration of splenic CD4^+^ T cells from naïve, D7 and D14 infected mice towards liver homogenate cells from (H) infected (D14) or (I) naïve mice. Data are the mean +/− SEM of the group with 3–5 mice per group. Data are representative of 3 independent experiments. *P<0.05, **P<0.01 WT vs. WSX-1^−/−^ mice.

To define the kinetics of altered intrahepatic accumulation of CD4^+^ T cells in WSX-1^−/−^ mice during malaria infection, we compared the frequencies and total numbers of intrahepatic CD4^+^ T cells in WT and WSX-1^−/−^ mice on various days of infection. No differences in either the frequencies or numbers of CD4^+^ T cells were observed in the livers of WT and WSX-1^−/−^ mice on days 4, 7 or 10 post-infection (PI) (Fig. 1A–C). By contrast, significantly increased frequencies and numbers of intrahepatic CD4^+^ T cells were observed in WSX-1^−/−^ mice on day 14 PI, at the time when they succumbed to infection (Fig. 1A–C and results not shown). As previously shown [3], WSX-1^−/−^, but not WT mice, developed severe liver necrosis and pathology on day 14 of infection, which was associated with accumulation of CD3^+^ lymphocytes within the tissue parenchyma proximal to central vein and sinusoids (Figure S1).

Ablation of WSX-1 signalling did not significantly affect the level of intrahepatic CD4^+^ T cell proliferation or apoptosis; intrahepatic CD4^+^ T cell expression of Ki67 and BrdU uptake was similar in WSX-1^−/−^ and WT mice and there were no differences in expression of Annexin V or 7-AAD on either day 7 or day 14 of infection (Fig. 1D–G). Combined, these data indicate that increased accumulation of CD4^+^ T cells in livers of WSX-1^−/−^ mice during malaria infection is not a result of dysregulated CD4^+^ T cell proliferation or apoptosis within the inflamed tissue.

### 2. Enhanced Migration of CD4^+^ T Cells from Malaria-infected WSX-1^−/−^ Mice towards the Inflamed Liver

We next hypothesised that increased CD4^+^ T cell accumulation within livers of WSX-1^−/−^ mice after day 10 of infection was due to active CD4^+^ T cell migration, most probably from the spleen, which is the major site of T cell priming during malaria infection [16]. Using an *in vitro* migration assay, we observed that very few CD4^+^ T cells from uninfected or day 7 infected WT or WSX-1^−/−^ mice or day 14 infected WT mice migrated across the transwell towards either naïve or infection-derived (D14) liver cells (Fig. 1H and results not shown). By contrast, CD4^+^ T cells from day 14-infected WSX-1^−/−^ mice exhibited significant migration towards day 14-derived liver cells (Figure 1H). Thus, CD4^+^ T cells acquire significant hepatotropic migratory capacity specifically in WSX-1^−/−^ mice only during the later stages of *P. berghei* NK65 infection. Significantly more CD4^+^ T cells from infected (D14 PI) WSX-1^−/−^ mice migrated towards liver cells from infected (D14 PI) WSX-1^−/−^ mice than migrated towards liver cells from infected (D14 PI) WT mice, indicating that factors produced by the infected liver also regulate CD4^+^ T cell migration, and that these signals differ between WT and WSX-1^−/−^ mice (Fig. 1H).

Migration of CD4^+^ T cells from infected (D14 PI) WT and WSX-1^−/−^ mice cells towards naïve liver cells and media was uniformly low (results not shown and Figure 1I), indicating that active, infection-induced, tissue signals were required to drive significant levels of CD4^+^ T cell migration. Thus, these data indicate that IL-27 represses the migratory capacity of splenic CD4^+^ T cells toward the inflamed liver during malaria infection and that both T cell-intrinsic and extrinsic factors appear to control CD4^+^ T cell chemotaxis.

### 3. Chemokine-chemokine Receptor Pathways are Dysregulated in WSX-1^−/−^ Mice during Malaria Infection

**Figure 2:**
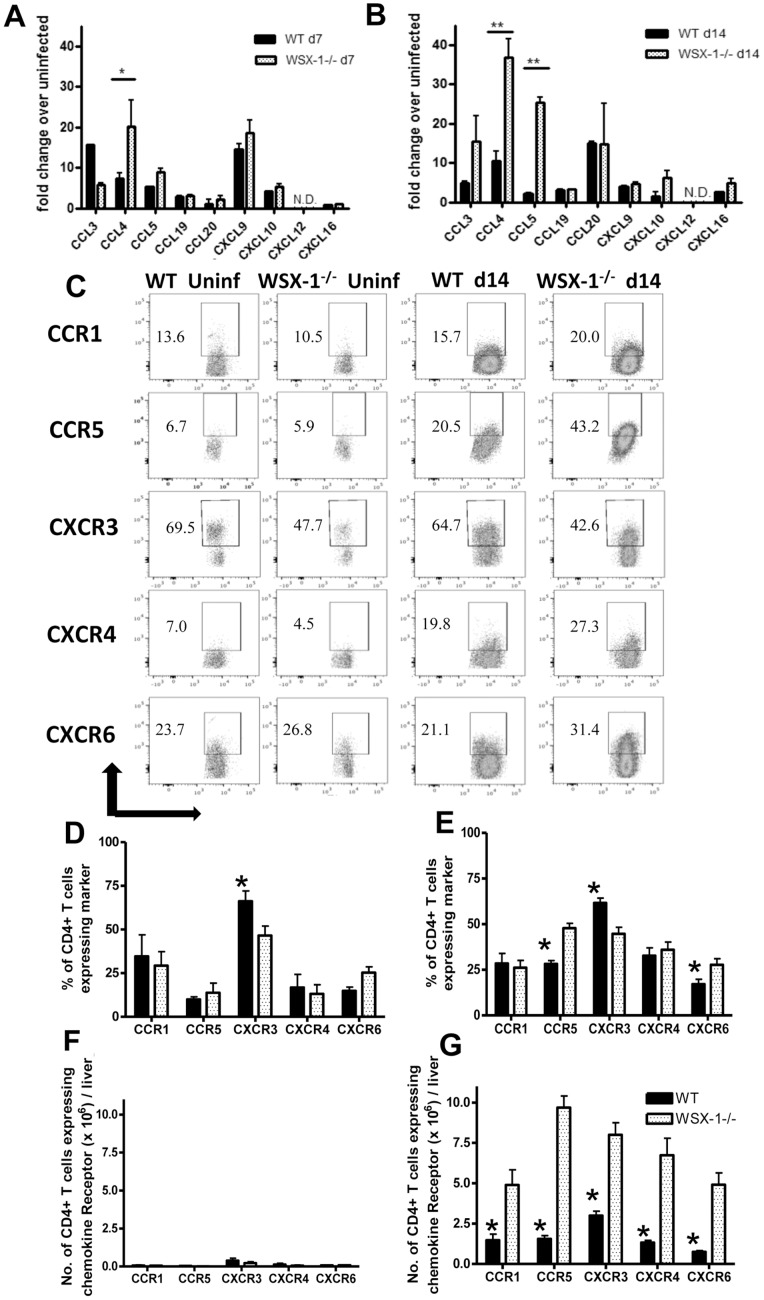
WSX-1 signalling restricts chemokine pathways in the liver during malaria-infection. (A, B ) Chemokine gene expression in the livers of WT and WSX-1^−/−^ mice on (A) day 7 and (B) day 14 of infection. (C) Representative dot plots of chemokine receptor expression by intahepatic CD4^+^ T cells from uninfected and infected mice (D14 PI). (D–G) The (D, E) Frequencies and (F, G) numbers of CCR expressing intra-hepatic CD4^+^ T cells from (D, F) uninfected and (E, G) infected (14 PI) WT and WSX-1^−/−^ mice. Data are the mean +/− SEM of the group with 3–5 mice per group. Data are representative of 3 independent experiments. *P<0.05, **P<0.01 WT vs. WSX-1^−/−^ mice.

To identify the soluble, WSX-1-regulated liver-derived signal(s) that control CD4^+^ T cell migration, we first compared the repertoire and levels of chemokine production in livers from WT and WSX-1^−/−^ mice on day 7 and day 14 of infection. Elevated levels of CCL3, CCL4, CCL5 and CXCL9 mRNA were detected in the livers of both WT and WSX-1^−/−^ mice on day 7 of infection, whereas CCL3, CCL4, CCL5 and CCL20 mRNA levels were increased on day 14 of infection. CCL4 was expressed at significantly higher levels in livers of WSX-1^−/−^ mice compared to WT mice on day 7 of infection (Fig. 2A), whereas CCL4 and CCL5, both ligands for CCR5, were expressed at 5–10-fold higher levels in livers of WSX-1^−/−^ mice compared to WT mice on day 14 of infection (Fig. 2B). IL-27R signalling therefore significantly and specifically represses production of CCL4 and CCL5 in the inflamed liver during malaria infection.

We next determined whether hepatotropic CD4^+^ T cells in infected WSX-1^−/−^ mice expressed a repertoire of chemokine receptors that enabled them to respond to the elevated CCL4 and CCL5 signals. Chemokine receptor expression among intrahepatic CD4^+^ T cells – with the exception of CCR5 - did not differ significantly between naïve and infected (D14 PI) mice (Fig. 2C–E). Interestingly, the proportion of intrahepatic CD4^+^ T cells expressing CCR5 was significantly higher in WSX-1^−/−^ mice than in WT mice on day 14 PI, whereas CXCR3 expression was higher among CD4^+^ T cells from WT mice (Fig. 2C–E). The majority of intrahepatic CD4^+^CCR5^+^ cells in infected WT and WSX-1^−/−^ mice co-expressed CXCR3, around 50% co-expressed CXCR6 and few cells co-expressed CCR1 (results not shown). In terms of absolute cell numbers, very few CD4^+^ T cells were found in the livers of naïve mice (Fig. 2F). In contrast, significantly higher numbers of CCR1^+^, CCR5^+^, CXCR3^+^, CXCR4^+^ and CXCR6^+^ T cells were found in the livers of WSX-1^−/−^ mice on day 14 of infection than in infected WT mice (Fig. 2G), due primarily to the significant increase in total CD4^+^ T cells accumulating in the livers of WSX-1^−/−^ mice during infection (Fig. 1A–C).

### 4. Altered Migration of CD4^+^ T Cells to the Liver in WSX-1^−/−^ Mice during Infection is not a Result of Dysregulated Chemokine Receptor Signalling

**Figure 3:**
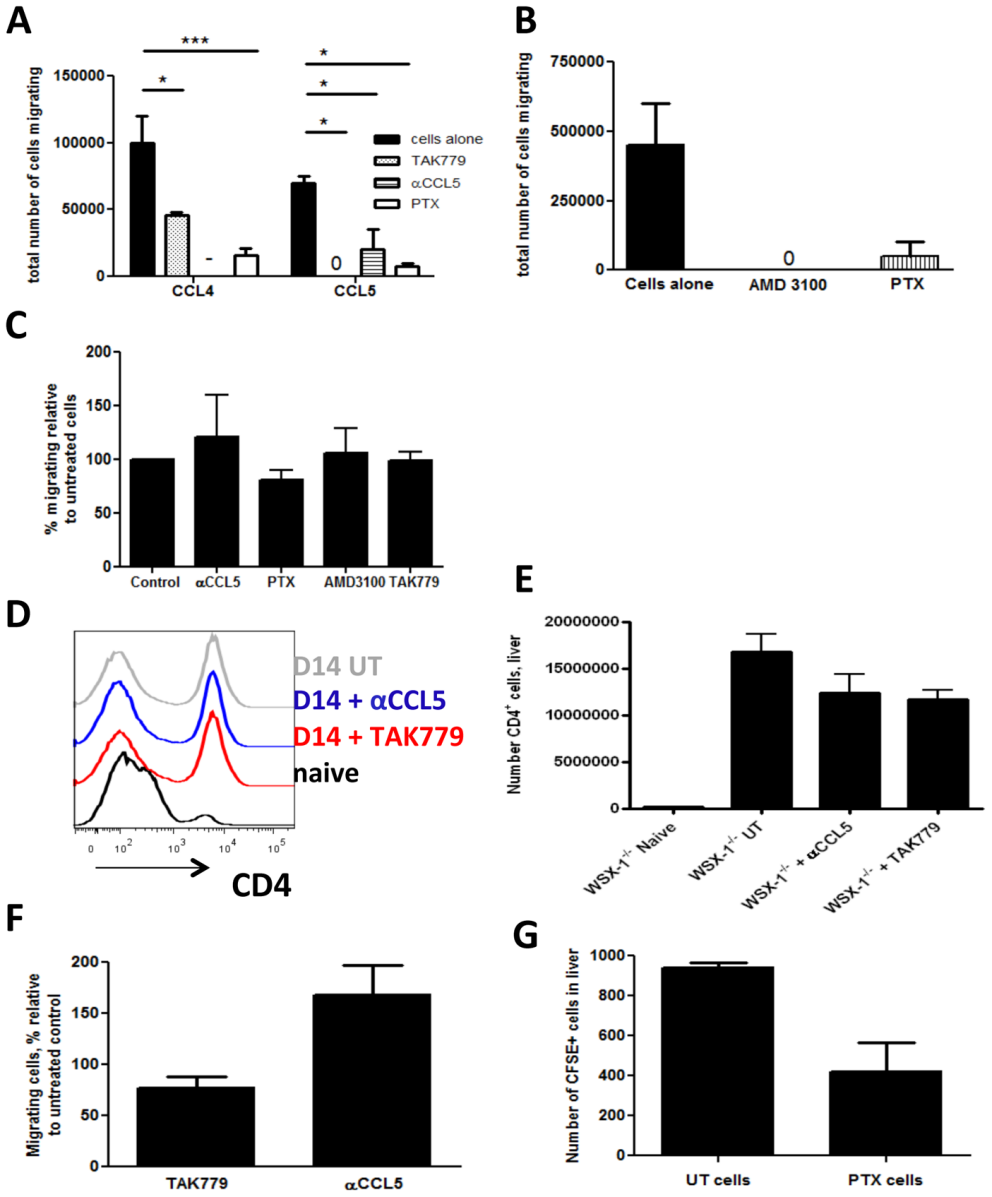
Abrogation of chemokine receptor signalling does not limit CD4^+^ T cell accumulation in the livers of WSX-1^−/−^ mice during malaria infection. The effect of inhibitors of chemokine receptor signalling on the migration of CD4^+^ T cells from infected (D14 PI) WSX-1^−/−^ mice towards (A) CCL4, CCL5, (B) CXCL12 and (C) liver homogenate cells from infected (D14 pi) WSX-1^−/−^ mice. (D) Representative histogram and (E) numbers of CD4^+^ T cells accumulating in the livers of treated and untreated WSX-1^−/−^ mice (D14 pi). (F) The migration of CD4^+^ T cells from treated infected (D14 PI) WSX-1^−/−^ mice towards liver homogenate cells from infected (D14 PI) WSX-1^−/−^ mice. (G) The numbers of pertussis toxin or control treated CD4^+^ T cells accumulating in the liver on day 13 pi. Data are the mean +/− SEM of the group with 3–5 mice per group. Data are representative of 3 independent experiments. *P<0.05 between highlighted groups.

The above data clearly demonstrate that IL-27 regulates the CCL4/CCL5-CCR5 axis within the liver during malaria infection. We postulated therefore that blocking CCL4/CCL5-CCR5 signalling should reduce migration to, and accumulation of, CD4^+^ T cells in the livers of WSX-1^−/−^ mice during infection. Surprisingly, although treatment with TAK779 (a CXCR3 and CCR5 antagonist), anti-CCL5 mAb, AMD3100 (inhibits CXCL12) and Pertussis toxin (PTX; inhibitor of all Gi protein coupled chemokine receptors) all significantly suppressed the chemotaxis of infection-derived, WSX-1^−/−^, CD4^+^ T cells towards the respective recombinant chemokines (Fig. 3A, B), none of these inhibitors prevented the migration of the purified CD4^+^ T cells towards infection-derived, WSX-1^−/−^ or WT, liver cells (Fig. 3C and results not shown for WT liver cells).

Consistent with the *in vitro* data, blockade of CXCR3/CCR5 by TAK779 and neutralisation of CCL5 both failed to suppress CD4^+^ T cell accumulation in WSX-1^−/−^ livers (Fig. 3D, E). CD4^+^ T cells removed from anti-CCL5- and TAK779-treated mice also exhibited unimpaired migration to infection-derived liver cells when placed in a transwell assay *in vitro* (Fig. 3F). In addition, and in agreement with the above data, PTX-treated CD4^+^ T cells were able to migrate normally to the livers of infected mice *in vivo* (Fig. 3G). These data show that although chemokine pathways are dysregulated in WSX-1^−/−^ mice during malaria infection, classical chemokine receptor pathways do not control CD4^+^ T cell accumulation within the liver.

### 5. Anti-IL-12p40 mAb Treatment Attenuates Th1 Polarisation and Suppresses Intrahepatic CD4^+^ T Cell Accumulation in WSX-1^−/−^ Mice during Malaria Infection

**Figure 4:**
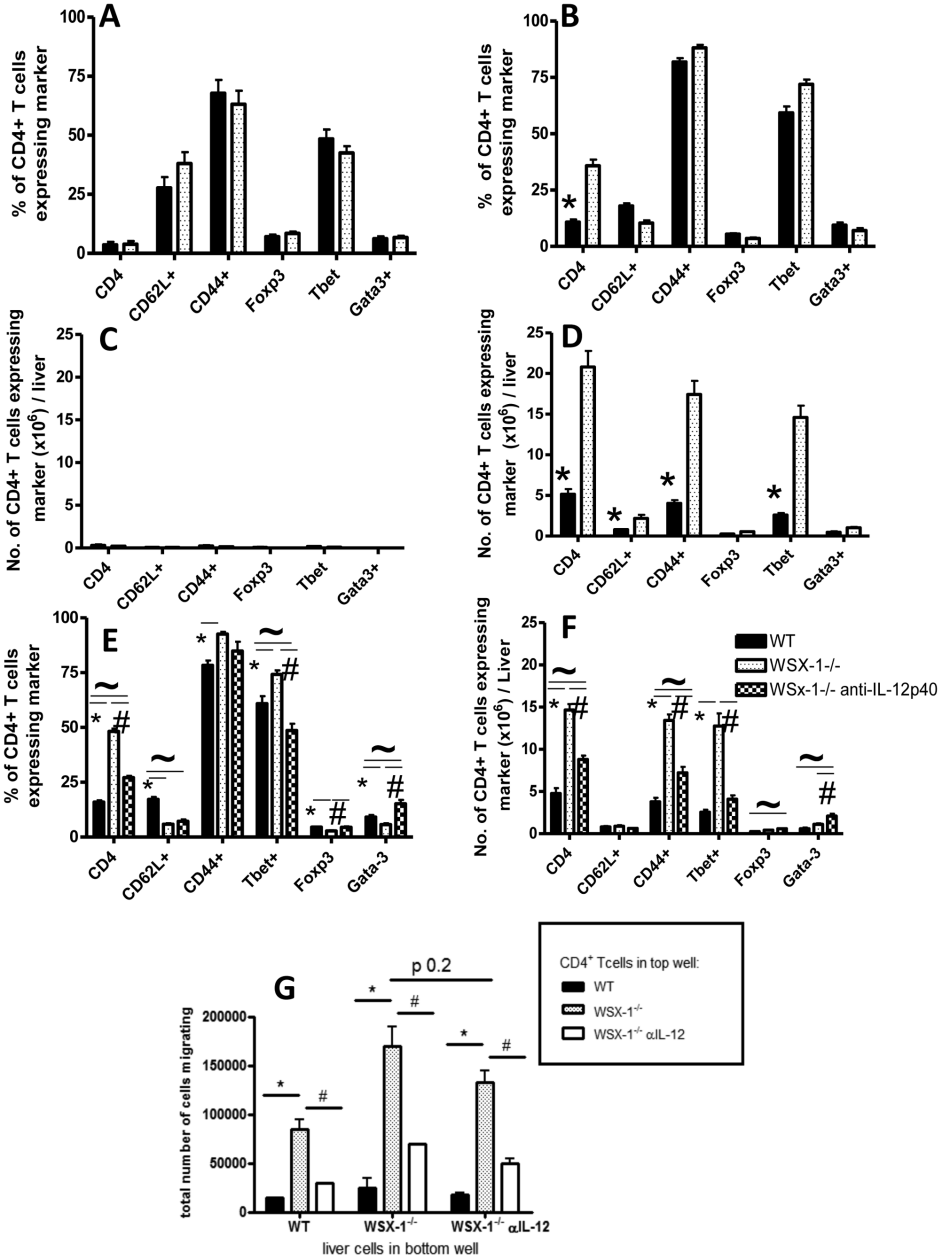
Neutralisation of IL-12 signalling reduces accumulation of pathogenic Th1 cells in the livers of WSX-1^−/−^ mice. The (A, B) frequencies and (C, D) numbers of intrahepatic CD4^+^ T cells from (A,C) uninfected and (B,D) infected (day 14 PI) WT and WSX-1^−/−^ mice expressing different activation and subset markers. (E–F) The effect of anti-IL-12p40 mAb administration on the (E) frequencies and (F) total numbers of liver accumulating CD4^+^ T cell subsets in WSX-1^−/−^ mice. (G) The migration of splenic CD4^+^ T cells from anti-IL-12p40 mAb treated and untreated mice towards liver homogenate cells from anti-IL-12p40 treated and untreated infected mice (D14 PI). Data are the mean +/− SEM of the group with 3–5 mice per group. Data are representative of 3 independent experiments. *P<0.05 WT vs. WSX-1^−/−^ mice; ^#^P<0.05 WSX-1^−/−^ vs. WSX-1^−/−^ + anti-IL-12 treatment. ∼P<0.05 WT vs. WSX-1^−/−^ + anti-IL-12 treatment.

The vast majority of CD4^+^ T cells in the livers of both naïve and infected, WT and WSX-1^−/−^ mice, were Th1 cells expressing both CD44 and T-bet (Fig. 4A, B). Consequently, significantly higher absolute numbers of CD44^+^ and T-bet^+^ CD4^+^ T cells accumulated within the livers of infected (but not naïve) WSX-1^−/−^ mice compared with WT mice (Fig. 4C, D). Thus, these data indicate that increased CD4^+^ T cell accumulation in the livers of WSX-1^−/−^ mice during infection is almost entirely driven by increased migration and/or retention of Th1 cells. As anti-IL-12p40 mAbs inhibit T-bet expression and Th1 cell differentiation in the spleen of malaria-infected WSX-1^−/−^ mice [14], where the hepatotropic cells likely originate (16), we hypothesised that suppressing Th1 responses in WSX-1^−/−^ mice by administering anti-IL-12p40 mAbs would significantly attenuate CD4^+^ T cell accumulation in the livers of WSX-1^−/−^ mice during malaria infection. As expected, anti-IL-12p40 mAb treatment of WSX-1^−/−^ mice from day 7 of infection onwards significantly reduced both the proportion and the absolute number of T-bet expressing CD4^+^ T cells accumulating in the liver (Fig. 4E, F).

CD4^+^ T cells from anti-IL-12p40 mAb-treated WSX-1^−/−^ mice showed reduced migration towards liver cells from (untreated) infected WSX-1^−/−^ mice compared with CD4^+^ T cells from infected but untreated WSX-1^−/−^ mice, indicating that anti-IL-12-mAb treatment significantly reduced CD4^+^ T cell migratory capacity (Fig. 4G). In addition, CD4^+^ T cells from both IL-12p40- mAb treated and untreated infected WSX-1^−/−^ mice showed slightly reduced (not-significant) migration towards liver cells from anti-IL-12-mAb treated mice. (Fig. 4G). Thus, IL-12 appears to play a major role in promoting CD4^+^ T cell migration to the liver of WSX-1^−/−^ mice during malaria infection, primarily by directing Th1 cell differentiation, rather than eliciting the production of soluble chemotactic signals from the inflamed liver.

## Discussion

In this study we have demonstrated that IL-27R/WSX-1 signalling is required to prevent the accumulation of large numbers of CD4^+^ T cells in non-lymphoid organs during the later stages of malaria infection. In so doing, IL-27 prevents the onset of potentially fatal liver pathology [3]. Surprisingly, we found that, compared with WT mice, there were no differences in the extent of *in situ* CD4^+^ T cell proliferation or apoptosis in the livers of WSX-1^−/−^ mice during infection. Rather, the aberrant accumulation of CD4^+^ T cells in the livers of malaria-infected WSX-1^−/−^ mice appears to be entirely driven by increased T cell migration: Splenic CD4^+^ T cells from malaria-infected WSX-1^−/−^ mice migrated towards liver homogenate cells, from either infected WT or WSX-1^−/−^ mice, significantly more efficiently than CD4^+^ T cells from infected WT mice. In addition, CD4^+^ T cells from infected WSX-1^−/−^ mice migrated towards liver homogenate cells from infected WSX-1^−/−^ mice significantly more efficiently than they did towards liver homogenate cells from infected WT mice. Thus, our results suggest that IL-27 simultaneously restricts the chemotactic capacity of CD4^+^ T cells, and also limits the production of soluble, liver-derived signals that direct T cell migration towards the inflamed tissue.

Intrahepatic CD4^+^ T cells from infected WSX-1^−/−^ mice expressed higher levels of CCR5 than corresponding cells from infected WT mice and, compared with infected WT mice, CCL4 and CCL5 were expressed at higher levels in the livers of infected WSX-1^−/−^ mice. As CCR1 and CCR5 play important roles in promoting lymphocyte recruitment to the liver during a variety of different inflammatory conditions [17], and as splenic CD4^+^ T cells from infected WSX-1^−/−^ mice are hyper-responsive to CCL4 and CCL5 [15], we initially expected that the enhanced migration to, and accumulation of, CD4^+^ T cells in the inflamed livers of infected WSX-1^−/−^ mice was driven by up-regulation of the CCR5-CCL4/CCL5 pathway. Surprisingly, however, blockade of chemokine receptor signalling using TAK779, AMD3100, anti-CCL5 mAb and pertussis toxin (blocking all Gi coupled receptors) did not prevent chemotaxis of CD4^+^ T cells from infected WSX-1^−/−^ mice towards infection-derived liver homogenate cells *in vitro,* suggesting that IL-27(R) inhibits CD4^+^ T cell migration to the liver via classical chemokine(R)-independent mechanisms. It is unlikely that there are technical explanations for these results as the treatments prevented the chemotaxis of infection-derived CD4^+^ T cells towards the relative recombinant chemokines, and pertussis toxin treatment inhibited chemotaxis towards all tested chemokines. Furthermore, chemokine pathway blockade failed to reduce accumulation of CD4^+^ T cells in the livers of infected WSX-1^−/−^ mice *in vivo.* Consequently, our results identify a novel IL-27(R) dependent pathway that limits CD4^+^ T cell migration to and accumulation within the liver during infection and inflammation.

Whilst we have been unable to identify the IL-27(R)-regulated tissue-derived soluble signals that promotes CD4^+^ T cell migration to the liver during infection, alternative chemoattractants that may control CD4^+^ T cell migration include High Mobility Group Box-1 (HMGB-1), a danger associated molecular pattern (DAMP) that induces neutrophil accumulation in the liver during viral infection [18], and which can act directly on T cells [19], [20], and VAP-1, which promotes T cell accumulation during ConA-induced liver inflammation [21]. The role of IL-27 in inhibiting DAMP pathways is yet to be examined in any model. Unexpectedly, however, anti-IL-12p40 treatment only partially suppressed the production of the chemotactic tissue-derived soluble signal(s) in infected WSX-1^−/−^ mice, despite preventing severe liver pathology [14]. Thus, the tissue migratory signal is not simply produced in the liver of infected WSX-1^−/−^ mice due to severe tissue damage. As well as directly targeting T cells, IL-27 may inhibit the production of chemotaxins in the liver during malaria infection by regulating the activation/function of macrophages/monocytes, neutrophils and hepatocytes [1], [2], [22].

Surprisingly the numbers of intrahepatic mast cells, basophils, neutrophils and resident monocytes/macrophages were unaltered in infected WSX-1^−/−^ mice and the numbers of inflammatory monocytes were decreased in WSX-1^−/−^ mice compared with WT mice (Figure S2). Consequently, the increased migration of CD4^+^ T cells to the liver of infected WSX-1^−/−^ mice does not appear to be driven by elevated intraheptic innate immune cell responses. Furthermore, although purified hepatic CD4^+^ T cells from infected (D14) WSX-1^−/−^ and WT mice, promote the migration of splenic CD4^+^ T cells from infected WSX-1^−/−^ mice, splenic CD4^+^ T cells do not display reduced migration towards liver homegenates obtained from infected mice treated with anti-CD4 mAbs (results not shown but provided for review). Thus, it does not appear that intrahepatic CD4^+^ T cells significantly contribute to production of the dominant tissue-chemotaxin(s) in WSX-1^−/−^ mice during infection.

Almost all the CD4^+^ T cells that migrated to, and accumulated within, the livers of infected WSX-1^−/−^ mice expressed T-bet and KLRG-1, marking them as terminally differentiated Th1 cells. The molecular pathways that govern the migratory capacity of terminally differentiated Th1 cells in this model are unclear. Th1 cells migrate very efficiently towards CXCR3 and CCR5 ligands [23], [24] but our data suggest that they also migrate towards non-classical, chemokine-independent signals emanating from inflamed liver cells. Although we have not defined the receptor that may control the migration of terminally differentiated Th1 cells in infected WSX-1^−/−^ mice, candidates include RAGE, the receptor for HMGB-1, which is expressed at higher levels on Th1 cells [25], and CD147, the cell membrane-expressed receptor for Cyclophilin A [26]. RAGE promotes leukocyte recruitment in a mouse model of peritonitis [27] and Cyclophilin A is released from necrotic liver cells [28] and can direct activated CD4^+^ T cell migration *in vitro* and *in vivo* [25], [29].

In summary, we have examined the mechanisms through which IL-27(R) signalling prevents pathological T cell accumulation in the liver during malaria infection. We have shown that IL-27 does not restrict CD4^+^ T cell proliferation or promote CD4^+^ T cell apoptosis *in situ* within the liver. Rather our results strongly indicate that IL-27 suppresses T cell migration to non-lymphoid tissues by limiting non-classical chemokine-independent pathways. Our results provide new information on the role of IL-27 in regulating T cell dependent immunopathology during inflammatory disease. We are currently examining the hypothesis that IL-27-regulated pathway(s) repress T cell accumulation in the liver by non-directly helping restrict the release of DAMPs from damaged hepatocytes and directly suppressing the expression of danger receptors on surveillent T cells.

## Supporting Information

Figure S1 **Histopathology of hepatic inflammation and tissue damage in malaria-infected WSX-1^−/−^ mice.** (A) Representative pictures showing the nature and level of hepatic pathology in malaria-infected WT and WSX-1^−/−^ mice. (B) Sections (D14) were examined by immunofluoescence following staining with DAPI, anti-CD3 and anti-CD45 antibodies. Data are representative of 2 independent experiments. Magnification 20×.
(PDF)

Figure S2 **Abrogation of WSX-1 signalling does not globally affect innate cell accumulation within the liver during malaria infection.** The absolute numbers of (A) mast cells: C-kit^+^IgE^+^FceR1^+^, (B) basophils: C-kit^-^FcEr1^+^, (C) inflammatory monocytes: CD11b^+^Ly6C^hi^Ly6G^low/int^ (D) neutrophils: CD11b^+^4-80^−^Ly6G^hi^Ly6Ci^nt/low^ (E) CD11b^−^F4-80^+^Ly6C^−^Ly6G^−^ and (F) CD11b^+^F4-80^+^Ly6C^−^Ly6G^−^ cells in the liver of naïve and malaria infected (D14 PI) mice. (E, F) cells represent resident monocytes and/or macrophages, including kupffer cells. All cellular populations were first gated from CD3- cells. Data are the mean +/− SEM of the group with 3–4 mice per group. Data are representative of 2 independent experiments. *P<0.05, WT vs. WSX-1^−/−^ mice.
(PDF)
