# Supplementary figures and images for: WSX-1 Signalling Inhibits CD4+ T Cell Migration to the Liver during Malaria Infection by Repressing Chemokine-Independent Pathways
Source: PLoS One. 2013 Nov 7;8(11):e78486. doi: 10.1371/journal.pone.0078486 (PMC3820588; doi:10.1371/journal.pone.0078486)

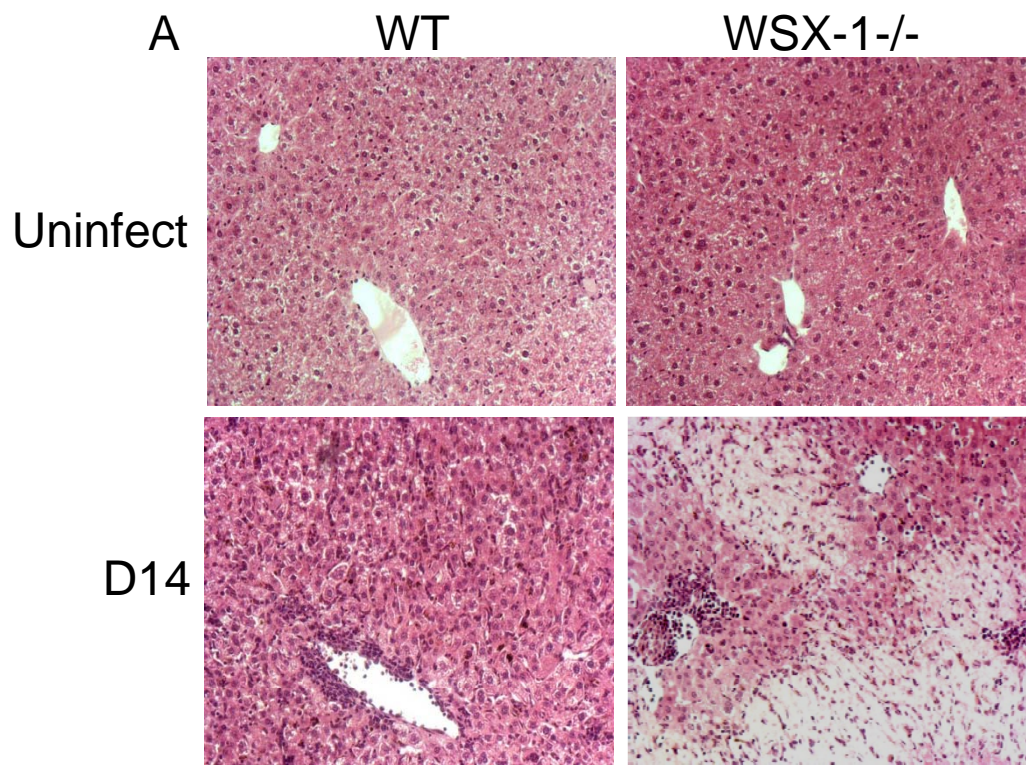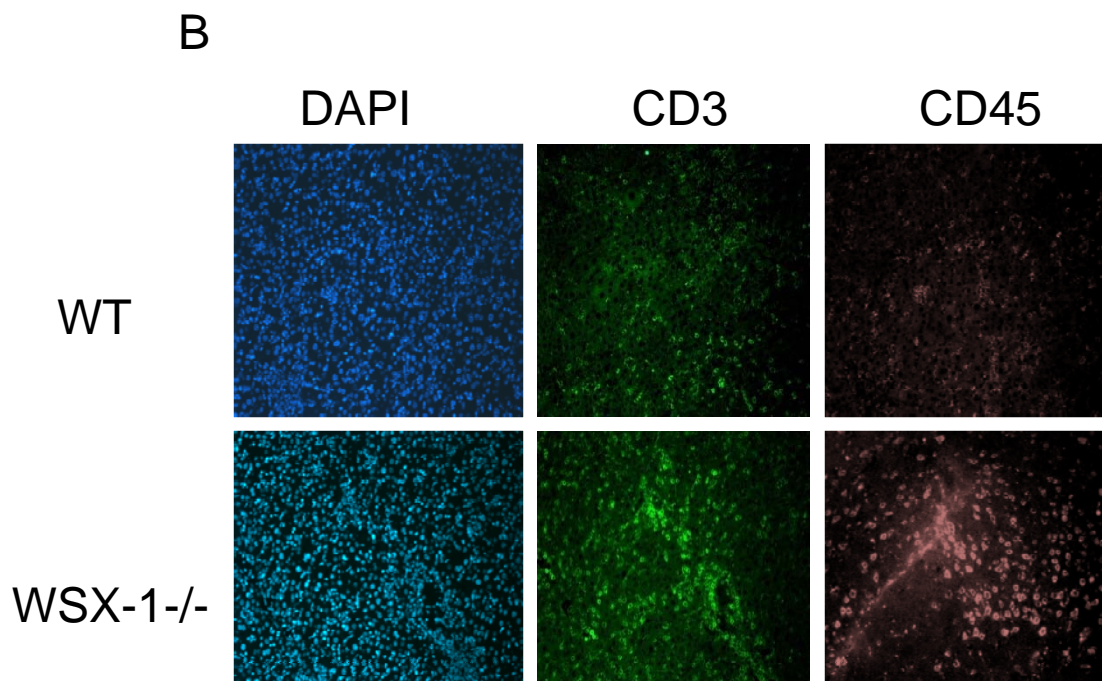

Supplement: Figure S1 — Histopathology of hepatic inflammation and tissue damage in malaria-infected WSX-1−/− mice. (A) Representative pictures showing the nature and level of hepatic pathology in malaria-infected WT and WSX-1−/− mice. (B) Sections (D14) were examined by immunofluoescence following staining with DAPI, anti-CD3 and anti-CD45 antibodies. Data are representative of 2 independent experiments. Magnification 20×. (PDF) [file pone.0078486.s001.pdf]

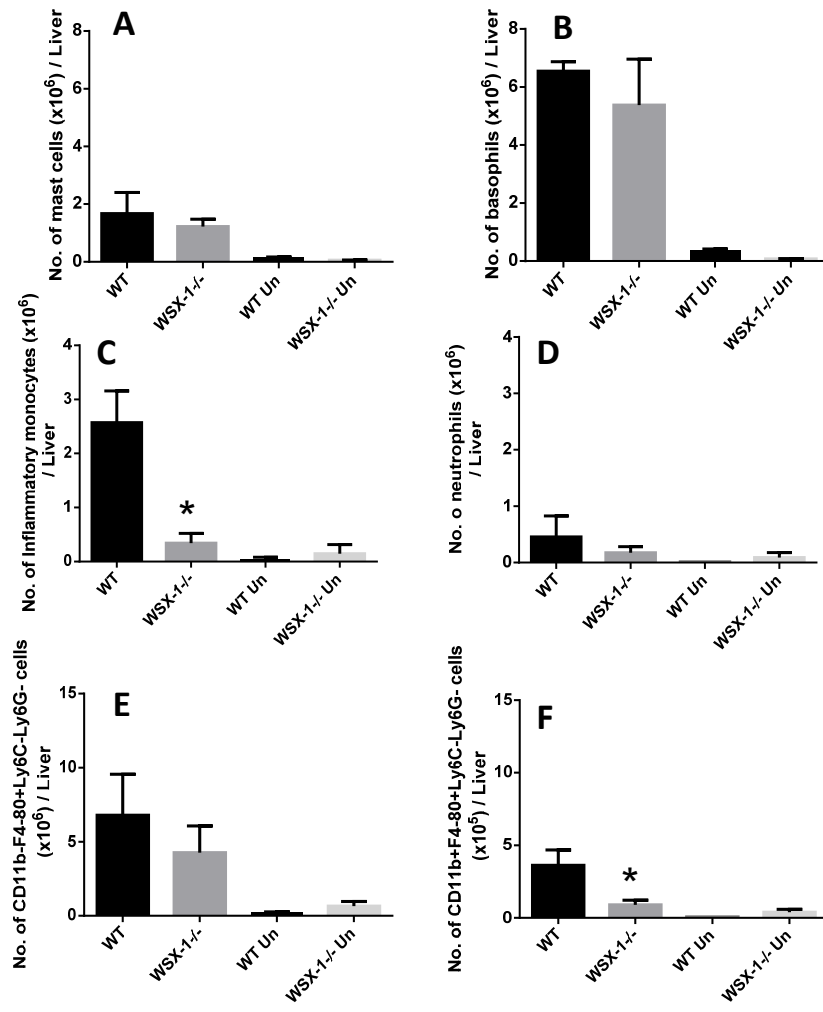

Supplement: Figure S2 — Abrogation of WSX-1 signalling does not globally affect innate cell accumulation within the liver during malaria infection. The absolute numbers of (A) mast cells: C-kit+IgE+FceR1+, (B) basophils: C-kit-FcEr1+, (C) inflammatory monocytes: CD11b+Ly6ChiLy6Glow/int (D) neutrophils: CD11b+4-80−Ly6GhiLy6Cint/low (E) CD11b−F4-80+Ly6C−Ly6G− and (F) CD11b+F4-80+Ly6C−Ly6G− cells in the liver of naïve and malaria infected (D14 PI) mice. (E, F) cells represent resident monocytes and/or macrophages, including kupffer cells. All cellular populations were first gated from CD3- cells. Data are the mean +/− SEM of the group with 3–4 mice per group. Data are representative of 2 independent experiments. *P<0.05, WT vs. WSX-1−/− mice. (PDF) [file pone.0078486.s002.pdf]
